# Accuracy of new intraocular lens calculation formulas in Chinese eyes with short axial lengths

**DOI:** 10.3389/fmed.2023.1257873

**Published:** 2023-10-10

**Authors:** Yueting Ma, Yongdong Lin, Yuancun Li, Zhuoyi Hu, Kunliang Qiu

**Affiliations:** Joint Shantou International Eye Center of Shantou University and The Chinese University of Hong Kong, Shantou, Guangdong, China

**Keywords:** short eyes, intraocular lens power, updated formula, phacoemulsification, refractive error

## Abstract

**Purpose:**

To compare the measurement accuracy of new/updated intraocular lens (IOL) power calculation methods, namely, Kane, Emmetropia Verifying Optical (EVO), with existing methods (Barrett Universal II, Olsen, Haigis, Hoffer Q, Holladay 1, SRK/T) in Chinese eyes with axial lengths ≤ 22.5 mm.

**Methods:**

The study included data from patients who underwent uneventful cataract surgery with the insertion of ZCB00 IOL. Refractive prediction errors were determined by calculating the difference between postoperative refraction and the predicted refraction using each formula. Various parameters were evaluated, including mean prediction error (ME), mean absolute error (MAE), median absolute error (MedAE), and the percentage of eyes with prediction errors (PE) within different ranges.

**Results:**

The study enrolled 38 eyes of 38 patients, and the Barrett Universal II formula demonstrated the lowest MAE and MedAE among the tested formulas. *Post hoc* analysis using Wilcoxon signed-rank pairwise comparisons for non-parametric samples with Bonferroni correction revealed no significant difference in postoperative refractive prediction among all the formulas (*P* > 0.05). The percentage of eyes with PE within ± 0.5 D was as follows: Barrett Universal II, 81.58%; Haigis, 78.95%; EVO, 76.32%; Olsen, 76.32%; Holladay I, 73.68%; SRK/T, 71.05%; Kane, 68.42%; and Hoffer Q, 65.79%.

**Conclusion:**

The Barrett Universal II formula was more accurate than the other formulas for Chinese eyes with AL ≤ 22.5 mm.

## Introduction

Advancements in optical coherent biometric measurement and the increasing use of phacoemulsification technology have significantly reduced the impact of measurement errors and surgical techniques on the outcomes of cataract surgery (1). Consequently, the accuracy and appropriate application of intraocular lens (IOL) calculation formulas have become crucial in determining the postoperative refractive state. Notably, accuracy tends to decrease in more complex eyes, such as those with short axial lengths (AL), long AL, and eyes with previous excimer laser surgery (2–4). Selecting the appropriate IOL power for patients with short ALhas proven to be particularly challenging, as demonstrated in previous studies (5, 6).

Third-generation IOL calculation formulas, such as Hoffer Q (7), SRK/T (8), and Holladay 1 (9), utilize AL and keratometry (K) data to determine effective lens position (ELP). Fourth-generation formulas, such as Haigis (10), add a parameter, preoperative anterior chamber depth (ACD), to the calculation of ELP. Fifth-generation formulas, including Barrett Universal II (11) and Olsen (12), consider additional parameters such as lens thickness (LT), ACD, and corneal white-to-white (WTW) in the ELP calculation. A study conducted by Melles et al. (11) involving 1,768 patients with short AL (< 22.5 mm), found that the Barrett Universal II formula exhibited the smallest prediction error compared to previous formulas.

The Kane formula, a recent addition, utilizes artificial intelligence and theoretical optics to predict IOL power. It requires AL, K, ACD, gender, and an A-constant, with the optional inclusion of LT and central corneal thickness (CCT) (13). The recently introduced Emmetropia Verifying Optical (EVO) formula requires AL, K, and ACD, with LT and CCT being optional parameters (14). Multiple studies showed that the new Kane formula demonstrated clear advantages in short eyes compared to previous formulas (6, 15, 16). The main population of these studies is white. However, a study conducted by Paritekar et al. (17) on Asian populations indicated that the new Kane formula did not exhibit distinct advantages in short eyes. To the best of our knowledge, few published studies assess the performance of the new Kane and EVO formulas in Asian populations with short eyes.

Therefore, the objective of this study was to compare the measurement accuracy of new/updated IOL power calculation methods (Kane, EVO) with existing methods (Barrett Universal II, Olsen, Haigis, Hoffer Q, Holladay 1, SRK/T) in Chinese eyes with AL of less than 22.5 mm.

## Materials and methods

This retrospective study was approved by the Ethics Committee of Joint Shantou International Eye Center and adhered to the tenets of the Declaration of Helsinki. The data for this study were collected from patients who underwent standard phacoemulsification and implantation of Tecnis monofocal ZCB00 IOL (Johnson & Johnson, New Jersey, USA) by experienced surgeons at the Joint Shantou International Eye Center between November 2017 and November 2019. Preoperative examinations, intraoperative events, and refractive data were recorded. Biometric parameters were obtained using the IOL Master 700 (Carl Zeiss AG, Germany) within 1 month prior to the surgery. Only eyes with AL ≤ 22.5 mm were included in the study, as described in the previous studies (11, 18–20). In cases where both eyes met the inclusion criteria, one eye was randomly selected for analysis.

The selection criteria followed the recommendations outlined in a recent editorial by Hoffer and Savini (21), which provided guidelines for best practices in IOL formula studies. Preoperative astigmatism was limited to 3.0 D or less, and a comprehensive manifest refraction examination was performed by a professional optometrist during the stable postoperative period, which ranged from 3 weeks to 1 month. Exclusion criteria encompassed additional surgical procedures performed during cataract surgery (e.g., peripheral corneal relaxing incisions), previous intraocular surgeries (including refractive corneal surgeries), intraoperative or postoperative complications, the presence of corneal pathology, significant fundus lesions impacting vision (e.g., diabetic retinopathy, retinal detachment, macular holes, macular epiretinal membranes), postoperative corrected distance visual acuity (CDVA) worse than 20/40, patients with nanophthalmos (AL < 20 mm), and incomplete subjective refraction data.

### Intraocular lens calculations

The optimization constants for the ZCB00 IOL in each formula were acquired from the User Group for Laser Interference Biometry (ULIB) website,^1^ as recommended by Hoffer and Savini (21) in a recent article. In accordance with the prerequisites of each formula, the values for gender, AL, keratometry, ACD, LT, CCT, and WTW measurements obtained via the IOL Master 700 were input. Each eye was entered independently (YL, ZH), and the results were cross-verified to prevent input errors. The formula calculators for Kane and EVO (V.2.0) were obtained from their respective official foreign websites.^2^^,^^3^ We did not zero out PE separately for the short AL group but instead applied the optimized A-constant for all AL ranges as described in the previous article (16, 22). The refractive prediction for each formula was calculated, and the difference between the calculated result and the actual postoperative refractive fraction was expressed as the equivalent spherical mirror. Mean prediction error (PE), mean absolute error (MAE), and median absolute error (MedAE) were calculated for each formula, and the percentage of eyes with PE within the ± 0.25 D, ± 0.5 D, ± 0.75 D, and ± 1.0 D ranges was recorded of each formula.

## Statistical analysis

We used SPSS (ver. 22.0; SPSS Inc., Chicago, IL, USA) analysis software for all statistical analyses. The Shapiro–Wilk test was performed to evaluate the normal distribution of continuous variable data. Differences in absolute error among formulas were evaluated using the Friedman test. In the event of a significant result, *post hoc* analysis was performed using the Wilcoxon test with Bonferroni correction. The Cochran Q test was used to compare the percentage of eyes with PE within ± 0.50 D of the actual outcome. *P* < 0.05 was statistically significant.

## Results

The study included a total of 38 eyes, with 22 right eyes and 16 left eyes, from 38 patients. The demographic characteristics of the eyes are presented in Table 1. Detailed data analysis, including refractive outcomes, MAE, and MedAE obtained by each formula and optimized constants for the ZCB00 IOL, can be found in Table 2.

**TABLE 1:** Characteristics of eyes in the study.

Parameter	Mean (± SD)
Age (year)	64.8 ± 7.52
Eye, n (%)	38
Right	22 (57.89%)
Left	16 (42.11%)
Average keratometry (D)	45.55 ± 1.08
Axial length (mm)	22.11 ± 0.37
Anterior chamber depth (mm)	2.86 ± 0.36
Lens thickness (mm)	4.54 ± 0.37
IOL Power (D)	24.89 ± 1.37

IOL, intraocular lens; D, diopter.

**TABLE 2:** Refractive prediction error, mean absolute error and median absolute error produced by each formula.

Formula	Optimized constants	PE	SD	MedAE	MAE	Percentage of Eyes within PE (%)			
	Tecnis ZCB00					≤0.25 D	≤0.50 D	≤0.75 D	≤1.00 D
Hoffer Q	5.8	−0.39	0.56	0.33	0.49	39.47%	65.79%	81.58%	84.21%
B U II	2.04	−0.06	0.54	0.24	0.37	50.00%	81.58%	81.58%	92.11%
EVO	119.3	−0.17	0.53	0.29	0.39	44.74%	76.32%	89.47%	89.47%
Haigis	−1.302	−0.07	0.56	0.27	0.38	44.74%	78.95%	84.21%	89.47%
	0.210								
	0.250								
Olsen	4.92	−0.08	0.63	0.30	0.44	39.47%	76.32%	84.21%	84.21%
Kane	119.36	−0.26	0.55	0.27	0.42	50.00%	68.42%	84.21%	89.47%
SRK/T	119.3	−0.31	0.51	0.39	0.45	36.84%	71.05%	81.58%	86.84%
Holladay I	2.02	−0.32	0.53	0.35	0.45	36.84%	73.68%	84.21%	86.84%

BU II, Barrett Universal II; EVO, Emmetropia Verifying Optical; PE, mean prediction error; SD, standard deviation of the error; MedAE, median absolute error; MAE, mean absolute error; D, diopter.

The results indicated that the Hoffer Q, Kane, SRK/T, and Holladay 1 formulas exhibited myopic prediction errors with mean deviations of −0.39 D, −0.26 D, −0.31 D, and −0.32 D, respectively (all *P* < 0.05). The Barrett Universal II formula demonstrated the lowest MAE of 0.37 and MedAE of 0.24. The Friedman test showed statistically significant differences in absolute prediction errors among the various formulas (*p* = 0.034). However, *post hoc* analysis using Wilcoxon signed-rank pairwise comparisons for non-parametric samples with Bonferroni correction revealed no significant differences in postoperative refractive prediction among the formulas (*P* > 0.05).

**FIGURE 1:**
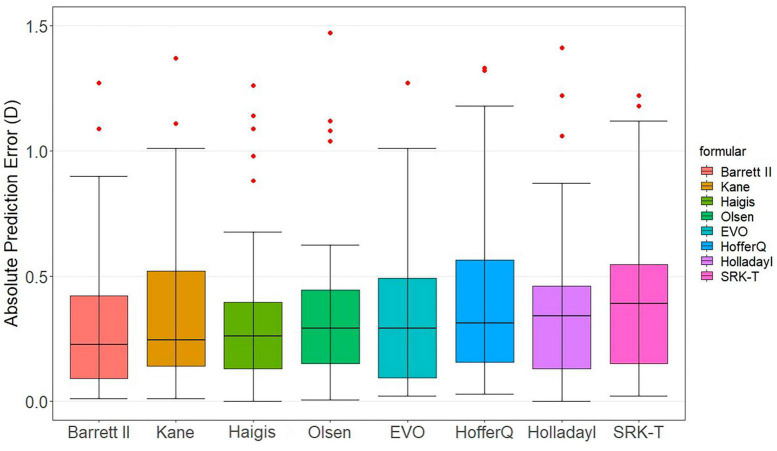
Distribution of the absolute prediction errors. Formulas are ranked according to the median absolute error, increasing from left to right. The central box represents the values from the lower to the upper quartile (25th to 75th percentile). The middle line represents the median.

Figure 1 presents the box-and-whisker plots and the distribution around the MedAE for each formula. All formulas demonstrated good outcomes. The distribution of the MedAE was found to be similar across all formulas, with little difference between the Barrett Universal II formula (lowest MedAE, 0.24 D) and the SRK/T formula (highest MedAE, 0.39 D). Interestingly, the classical Haigis formula outperformed the newer, last-generation formulas, namely, EVO and Kane. This observation was corroborated by the box chart, which illustrated that the deviation of the Haigis formula was the most modest. Consequently, Haigis (MedAE 0.27 D) exhibited superior performance compared to Kane (MedAE 0.27 D) and EVO (MedAE 0.29 D).

**FIGURE 2:**
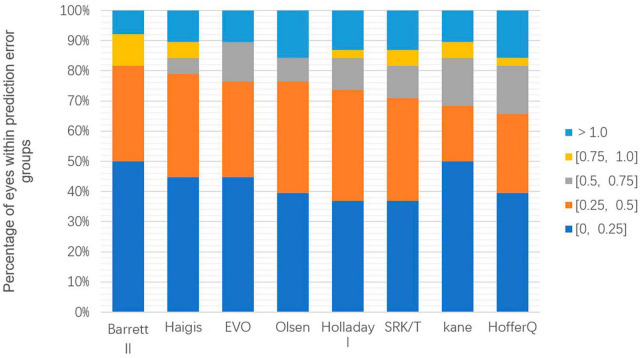
Stacked histogram comparing the percentage of cases with a given absolute prediction error. Formulas are ranked according to the higher percentage for the prediction error within ± 0.50 diopters.

Figure 2 displays the percentage of eyes within a certain PE, ranked according to the highest percentage within ± 0.50 D. Cochran’s Q test results indicated that the proportion of eyes with a PE within ± 0.50 D did not differ significantly among the various formulas (*p* = 0.241) among the formulas. The range varied from 65.79% (Hoffer Q) to 81.58% (Barrett Universal II) of eyes with PE within ± 0.50 D. Only the Barrett Universal II and Kane formulas achieved 50% of eyes with PE within ± 0.25 D.

## Discussion

To the best of our knowledge, this is the first study to evaluate the performance of the Kane, EVO, and Barrett Universal II formulas in the Chinese population with short eyes. In our cohort, the Barrett Universal II formula demonstrated the highest accuracy, with the lowest MAE, MedAE, and the highest percentage of eyes with PE within 0.25 D, 0.50 D, and 1.00 D.

Despite the continuous updates and diversity of formulas, there are still limitations in the accuracy of lens calculations for short eyes. Several studies showed the Barrett Universal 2 formula outperformed third-generation and Haigis formulas, which was consistent with the findings of our study (11, 15, 23). The Hoffer Q formula exhibited the largest prediction error and MAE in our study, contradicting some early studies that suggested its reliability in short eyes (24). Wang et al. (25) conducted a meta-analysis of 10 observational studies (1,161 eyes) to evaluate the accuracy of different formulas (Hoffer Q, SRK II, SRK/T, Holladay I, Holladay II, and Haigis) in calculating the IOL power of eyes with AL < 22 mm. Their results showed that Haigis formula (MAE: 0.498) was more accurate than Hoffer Q (MAE: 0.510), SRK/T (MAE: 0.555), and SRK II (MAE: 1.146), which aligned with our findings. Our research indicated that the MAE of the Haigis formula was lower than that of the third-generation formula and new formula (Kane, EVO), but higher than that of the Barrett Universal II formula. The Barrett Universal II formula demonstrated the lowest MAE of 0.37. The third-generation formulas, such as Holladay 1, SRK/T, and Hoffer Q, showed lower accuracy in predicting refraction, possibly due to the limited number of biometric parameters they utilize, relying solely on AL and K readings to calculate the ELP (25).

The Kane formula, developed based on artificial intelligence and theoretical calculations, has gained popularity in recent years (26). In addition to gender, the Kane formula incorporates five variables: AL, K, ACD, LT, and CCT. The results are further optimized using artificial intelligence. Recent studies have demonstrated that the Kane formula is significantly more accurate than other formulas in calculating IOL power for short eyes (AL ≤ 22.00 mm) (6, 16). A large-scale study involving 766 eyes and four different IOLs, using a partial coherence interferometry (PCI) biometer, found that the Kane formula had the lowest MAE of 0.441 in short eyes (AL ≤ 22.00 mm) (15). Connell and Kane’s study did not find any statistically significant differences among the absolute PE of the formulas in the short AL group (*n* = 46) (26). The Kane (MAE 0.441 D) formula was the most accurate predictor of postoperative refractions in their study. However, research by Paritekar et al. (17) on Indian populations demonstrated that the Kane formula did not exhibit significant advantages in short AL eyes, which was consistent with our findings. In our study, the Kane formula also did not show clear advantages in short eyes. This could be attributed to differences in the implanted IOLs. The accuracy ranking of the formulas can vary depending on the specific IOL model used (15). All IOL formulas are based on data derived from clinical practice. In our study, the same IOL type was used as in the study by Paritekar et al. (17). Moreover, the differences in racial characteristics may have contributed to the discrepancy. Most previous studies on the Kane formula focused on Caucasian populations.

The EVO formula is a new thick lens formula based on the concept of emmetropization. In our study, the EVO formula performed reasonably well, with 76.32% of eyes with PE within ± 0.5 D, ranking third after the Barrett Universal II and Haigis formulas. However, the performance of the EVO formula was inferior in both short and long AL eyes, as observed in the study by Melles et al., suggesting that the concept of emmetropization might not be as effective at the extremes of AL (13).

Intraocular lens calculations for short eyes are notably less accurate compared to the overall patient population. This is primarily due to the magnification of any errors in AL measurement or effective lens position estimation by the higher dioptric power of the IOL (3). In our study, the Barrett Universal II formula demonstrated good performance, with the highest percentage (81.58%) of eyes with PE within ± 0.5 D, followed by the Haigis (78.95%) and EVO 2.0 (76.32%) formulas. These findings differ from those of Gökce et al. (5). Barrett Universal II formula in their research gave less than 70% results. This discrepancy may be attributed to the shorter AL in their study, with an average of 21.53 mm, compared to the average AL of 22.11 mm in our study. Additionally, differences in the racial populations studied have been shown to contribute to variances in biological eye characteristics (27). We also observed that due to the limited sample size, no patients fell within the range of PE between 0.5 and 0.75 D in the Barrett Universal II formula. Addressing these outliers will necessitate an expansion of the sample size in future studies. The results in our study align with those of Melles et al. (11). Barrett Universal II formula in their research had the 80.0% percentage for the SA60AT IOL. Our findings suggest that there were modest differences between the formulas tested, with refractive prediction errors of approximately 0.32 D on the myopic side with the Hoffer Q, Kane, SRK/T, and Holladay 1 formulas. Similar findings were reported by Paritekar et al. (17) in their study. Thus, the recommended lens constant for the Tecnis IOL should be altered for optimization.

Our study has some limitations. Firstly, the inclusion of data from multiple surgeons may introduce bias due to differences in surgical techniques. However, it has been demonstrated that in modern surgery and optometry, this has minimal impact on the results (2, 15). Secondly, our sample size was small, which may have somewhat reduced the statistical power and confidence in our analyses, and our study only included one type of IOL and individuals of Chinese ethnicity. As a result, the generalizability of our findings to other intraocular lens models and different racial or ethnic groups may be limited. Moving forward, we plan to enroll a larger number of patients and include different IOL types to increase the reliability and generalizability of our research. Lastly, we did not personally optimize the constants used. Hoffer and Savini (21) have advocated for relying on optimization constants derived from the entire population rather than constants specifically calculated for subsets like short-eye samples when analyzing short eyes (21). Additionally, they also advocated that it was considered acceptable to employ optimization constants (for each IOL) provided by extensive databases, such as ULIB, in the study. Given our relatively small sample size, Haigis (personal communication, 2010) suggests that to achieve reliable optimization constant measurements, at least 100 eyes should be included. Consequently, the optimization constants utilized in our study were drawn from the overall population data available in the ULIB database.

## Conclusion

In conclusion, our study found that the Barrett Universal II formula was more accurate than the other formulas for Chinese eyes with AL ≤ 22.5 mm. The Hoffer Q, Kane, SRK/T, and Holladay 1 formulas exhibited slightly myopic refractive prediction errors.

## Data availability statement

The original contributions presented in this study are included in this article/supplementary material, further inquiries can be directed to the corresponding author.

## Ethics statement

The studies involving humans were approved by the Ethics Committee of Joint Shantou International Eye Center (JSIEC) of Shantou University and the Chinese University of Hong Kong (Shantou city, China). The studies were conducted in accordance with the local legislation and institutional requirements. The Ethics Committee/institutional review board waived the requirement of written informed consent for participation from the participants or the participants’ legal guardians/next of kin because this study was retrospective, informed consent for inclusion was waived.

## Author contributions

YM: Conceptualization, Data curation, Methodology, Writing–original draft. YDL: Conceptualization, Data curation, Methodology, Writing–original draft. YCL: Data curation, Software, Writing–original draft. ZH: Data curation, Writing–original draft. KQ: Funding acquisition, Writing–original draft, Writing–review and editing.

## Footnotes

1
www.ocusoft.de/ulib/c1.htm

2
https://www.iolformula.com

3
https://www.evoiolcalculator.com
